# Exploring Relationships of Heart Rate Variability, Neurological Function, and Clinical Factors with Mortality and Behavioral Functional Outcome in Patients with Ischemic Stroke

**DOI:** 10.3390/diagnostics14121304

**Published:** 2024-06-20

**Authors:** Mei-Jung Wu, Sari R. K. Dewi, Wan-Ting Hsu, Tien-Yu Hsu, Shu-Fen Liao, Lung Chan, Ming-Chin Lin

**Affiliations:** 1 Graduate Institute of Biomedical Informatics, College of Medical Science and Technology, Taipei Medical University, New Taipei City 235, Taiwan; 2 Nursing Department, Wan Fang Hospital, Taipei Medical University, Taipei 116, Taiwan; 3 Institute of Brain Science, National Yang Ming Chiao Tung University, Taipei 112, Taiwan; 4 Sleep Research Center, National Yang Ming Chiao Tung University, Taipei 112, Taiwan; 5 Department of Medical Research, Wan Fang Hospital, Taipei Medical University, Taipei 116, Taiwan; 6 School of Public Health, College of Public Health, Taipei Medical University, New Taipei City 235, Taiwan; 7 Department of Neurology, Shuang Ho Hospital, Taipei Medical University, New Taipei City 235, Taiwan; 8 Department of Neurology, School of Medicine, College of Medicine, Taipei Medical University, Taipei 110, Taiwan; 9 Taipei Neuroscience Institute, Taipei Medical University, Taipei 110, Taiwan; 10 Department of Neurosurgery, Wan Fang Hospital, Taipei Medical University, Taipei 116, Taiwan; 11 Department of Neurosurgery, Shuang Ho Hospital, Taipei Medical University, New Taipei City 235, Taiwan

**Keywords:** heart rate variability, ischemic stroke, functional outcome, mortality, neurological function

## Abstract

Ischemic stroke is a leading cause of mortality and disability. The relationships of heart rate variability (HRV) and stroke-related factors with mortality and functional outcome are complex and not fully understood. Understanding these relationships is crucial for providing better insights regarding ischemic stroke prognosis. The objective of this study is to examine the relationship between HRV, neurological function, and clinical factors with mortality and 3-month behavioral functional outcome in ischemic stroke. We prospectively collected the HRV data and monitored the behavioral functional outcome of patients with ischemic stroke. The behavioral functional outcome was represented by a modified Rankin Scale (mRS) score. This study population consisted of 58 ischemic stroke patients (56.9% male; mean age 70) with favorable (mRS score ≤ 2) and unfavorable (mRS score ≥ 3) outcome. The analysis indicated that the median of the mean RR interval (RR mean) showed no statistical difference between mortality groups. Conversely, the median of the RR mean had significant association with unfavorable outcome (OR = 0.989, *p* = 0.007). Lower hemoglobin levels had significant association with unfavorable outcome (OR = 0.411, *p* = 0.010). Higher National Institute of Health Stroke Scale (NIHSS) score at admission had significant association with unfavorable outcome (OR = 1.396, *p* = 0.002). In contrast, age, stroke history, NIHSS score at admission, and hemoglobin showed no significant association with mortality in ischemic stroke. These results imply that HRV, as indicated by the median of RR mean, alongside specific clinical factors and neurological function at admission (measured by NIHSS score), may serve as potential prognostic indicators for 3-month behavioral functional outcome in ischemic stroke.

## 1. Introduction

Stroke is a neurologic disorder that is fast becoming the second leading cause of death and long-term disability worldwide. Ischemic stroke is the most common form of stroke, with approximately 87% of prevalence [1]. Between 1990 and 2019, ischemic stroke incidence and deaths increased > 60% [2]. The number of the disability-adjusted life years (DALYs) attributed to ischemic stroke increased by 56.72% [3]. Yet, disability and function loss commonly happen in stroke survivors and impact their quality of life, an element that is influenced by multiple factors [4]. Therefore, understanding the association between the ischemic stroke outcome-related factors with mortality and functional outcome is important for providing the basic evidence of stroke prognosis to support stroke treatment strategies and optimize stroke management. 

Dysfunction of the autonomic nervous system (ANS) has been reported in ischemic stroke because the reduction in cerebrovascular blood flow (CBF) leads to subsequent neuronal dysfunction and potentially irreversible tissue damage. The ANS dysfunction also contributes to the risk in the pre-pathological state and determines clinical complications with poor outcome [5]. The non-invasive measurement of autonomic dysfunction has been established and used widely by measuring cardiac autonomic function using heart rate variability (HRV) [6].

HRV is the fluctuation in the time interval between consecutive heartbeats generated by heart–brain interactions and dynamic non-linear ANS. HRV can be used for ANS measurement by describing the HRV using linear frequency-domain, linear time-domain, or non-linear measurement. The previous studies showed the various results of the correlation between the HRV parameters and ischemic stroke functional outcome, including neurological, behavioral, cognitive, and emotional function. Most studies showed that lower HRV parameters of all domain types were correlated with higher severity, poor neurological function, and poor behavioral outcome in ischemic stroke [7]. In contrast, Hilz et al. found that a higher low-frequency (LF) and LF/high-frequency (HF) ratio were correlated with higher stroke severity [8]. Studies by Tang et al. and Tsai et al. showed no difference in HRV between the patients with poor and good behavioral functional outcome evaluated by using a modified Rankin Scale (mRS) score in regard to ischemic stroke [9,10]. The existing previous studies showed that the correlation between HRV with stroke severity and functional outcome is still inconsistent and complicated in regard to HRV complexity. Consequently, the purpose of this study is to assess how HRV parameters, neurological function, and clinical factors are associated with mortality and the behavioral functional outcome at 3 months. 

## 2. Materials and Methods

### 2.1. Study Design and Population

The protocol was approved by the Institutional Review Board of Taipei Medical University to collect the data in this study. Conducted between April 2019 and May 2020, this study involved a two-step selection process. In the first step, patients admitted to the neuro intensive care unit (NCU) who agreed to participate were enrolled. Exclusion criteria at this stage included patients who declined participation, exhibited specific clinical conditions (such as wounds, unconsciousness, irritability, thin or irritated skin, restlessness, cognitive impairment, or drooling), were placed in an isolation room, or required medical equipment (including endotracheal tubes, tracheal tubes, ventilators, or neck collars). All enrolled patients provided informed consent to participate in this study. In the second step, only patients diagnosed with ischemic stroke and admitted to NCU within 24 h were selected for this study. They were monitored until discharge, with follow-up assessments being conducted at 3 months post-admission. We excluded patients who failed to undergo HRV analysis or were not diagnosed with ischemic stroke. Evidence of ischemic lesions was determined by neuroimaging studies, such as computerized tomography (CT) scans or magnetic resonance imaging (MRI). In this study, the information of ischemic stroke patients was collected, including demographics (age and gender), disease history, body mass index (BMI), length of stay, mortality status, and infarct side. The infarct side information was determined from the neuroimaging report.

### 2.2. Neurological and Behavioral Function Assessment

We did two outcome-related assessments, including neurological function and behavioral function. Neurological function was evaluated by using the National Institutes of Health Stroke Scale (NHISS) for evaluating stroke severity. The NIHSS scores were collected upon admission and discharge. Behavioral function, as the functional outcome in this study, was scored using the mRS score. The mRS is widely recognized and the standard tool for assessing functional outcome in stroke patients due to its simplicity and reliability in measuring disability levels. It is categorized into seven levels, with 0 indicating no symptoms, 3 representing moderate disability, 5 indicating severe disability, and 6 indicating death [11].

In this study, the functional outcome of the patients were categorized into two groups based on their mRS scores, consisting of favorable outcome (mRS score 0–2) and unfavorable outcome (mRS score 3–6) [9]. Previous studies reported stroke functional outcome across multiple function domains, including cognitive, emotional, and behavioral [7]. However, for the purpose of this study, we specifically defined the functional outcome within the behavioral function domain as the behavioral functional outcome. Additionally, the statistical analysis of the functional outcome only included the long-term behavioral functional outcome, which were determined by mRS scores at 3 months.

### 2.3. HRV Measurement

**ECG Signal Acquisition and Processing.** The ECG signal acquisition and processing followed the previous publication from Kuo et al. [12]. The ECG signal was collected directly from the ischemic stroke patient as soon as possible after the patient was admitted to the neuro ICU. The ECG signals were extracted and digitized at a sampling rate of 125 Hz and saved on a computer. The digitized ECG signal was processed to extract the RR interval before further HRV analysis. The RR interval was defined as the interval between two time points of each heartbeat (R point) of each valid QRS complex.

**HRV Analysis.** We performed frequency-domain analysis using the nonparametric method of Fourier transform (FFT). The detailed steps of analysis were also previously published. The analyzed frequency-domain HRV parameters included TP, LF, HF, LF/HF ratio, and LF%. The powers in each HRV range category in ms^2^ were defined as LF and HF. On the other hand, we also normalized LF by the percentage of total power, excluding VLF (total power—VLF), into LF power in normalized units (LF%). Additionally, we examined a time-domain HRV parameter, the RR mean, which is defined as the average RR interval [12]. In this study, the analysis of the relationship between HRV and behavioral functional outcome also only focused on the 3-month mRS score.

### 2.4. Statistical Analysis

The LF, HF, and LF/HF were logarithmically transformed, as referred to in the previous study [12]. Then, all HRV parameters in this study were expressed as the median. Statistical analysis in this study was performed by IBM SPSS software version 19 (IBM Corporation, Armonk, NY, USA). We performed an independent *t*-test, Mann–Whitney U test, and chi-square χ2 test to compare the demographic, BMI, risk factor, NIHSS at admission, HRV parameter, and infarct side for the functional outcome group and mortality status. Logistic regression analyses were performed for the significant variables in comparison analysis (*p* < 0.05) by calculating the crude odds ratio and adjusted odds ratio with a 95% confident interval. The potential prognostic indicator was assigned by *p*-value < 0.05 in multivariable logistic regression analyses.

## 3. Results

A total of 337 consecutive patients who were admitted to the NCU, Shuang Ho Hospital, agreed to participate in this study. We excluded 279 patients who were not diagnosed with ischemic stroke. Fifty-eight selected patients underwent the HRV analysis after admission and evaluation of NHISS at admission and discharge. 

### 3.1. Baseline Characteristic

A total of 58 ischemic stroke patients were included in the analysis (33 male, mean age 70 years). The obese patient proportion (53.4%) in this study is higher than non-obese patients. At admission, 27 patients (46.6%) had a moderate stroke, 20 patients (34.5%) had a severe stroke, and 11 patients had a mild stroke (18.9%). At discharge, the number of patients with mild stroke increased to 25 (43%). Also, among the 58 ischemic stroke patients, 45 patients (77.6%) survived. The detailed characteristics of all included patients can be seen in Table 1. 

### 3.2. Association of HRV Parameter, Neurological Function, Clinical Factors with Mortality

In Table 2, no significant differences were identified in both HRV parameters (time-domain and frequency-domain parameters) between the survival and death groups (all *p*-values > 0.05). The NIHSS scores at admission were significantly lower (mean score of 11) in the survival group than in the death group (*p*-value = 0.003). In Table 3, the stroke survivors were significantly younger (mean age 68 years) compared to those who did not survive at 3 months (*p*-value = 0.029), while a history of stroke was significantly more prevalent in the death group than in the survival group (*p*-value = 0.004). Hemoglobin (Hb) concentration also showed a significant difference between the survival and death groups (*p*-value = 0.044). In contrast, no significant differences were observed in the comparison of the infarct side and BMI in regard to mortality status (all *p*-values > 0.05).

Four significant variables identified through comparison analysis (Table 2 and Table 3) were included in the univariate and multivariable logistic regression analysis (Table 4), including NIHSS score at admission, age, stroke history, and hemoglobin. These variables showed consistent effects based on the odds ratio in both logistic regression analyses (NIHSS score at admission: univariate OR = 1.134, adjusted OR = 1.109; age: univariate OR = 1.068, adjusted OR = 1.024; stroke history: univariate OR = 0.743, adjusted OR = 0.267; Hb: univariate OR = 0.111, adjusted OR = 0.835). However, in the multivariable logistic regression analysis, none of these variables were found to be significantly associated with mortality in ischemic stroke patients, as all *p*-values were greater than 0.05 (NIHSS score at admission: adjusted OR = 1.109, 95% CI = 0.988–1.244, *p*-value = 0.079; age: adjusted OR = 1.024, 95% CI = 0.944-1.111, *p*-value = 0.570; stroke history: adjusted OR = 0.267, 95% CI = 0.041–1.736, *p*-value = 0.167; hemoglobin concentration: adjusted OR = 0.835, 95% CI = 0.565–1.235, *p*-value = 0.366).

### 3.3. Association of HRV Parameters, Neurological Function, and Clinical Factors with Behavior Functional Outcome

At the 3-month follow-ups, only the median of the RR mean was found to be significantly different in terms of HRV parameters when compared with behavioral functional outcome (*p*-value = 0.049, Table 5). Specifically, the median of the RR mean in the favorable group was significantly higher than in the unfavorable group. Conversely, there were no differences observed in the frequency-domain HRV parameters between the favorable and unfavorable groups (all *p*-values > 0.05, Table 5). Additionally, the NIHSS score at admission was significantly lower in the favorable group than the unfavorable group (*p*-value ≤ 0.0001, Table 5). The proportion of stroke patients with a history of stroke was significantly lower in the favorable group (3.6%, Table 6) compared to the unfavorable group (*p*-value = 0.015, Table 6). Furthermore, Hb concentration and eGFR at admission were significantly higher in the favorable group than in the unfavorable group (*p*-value of Hb = 0.018 and *p*-value of eGFR = 0.022). For further comparisons of clinical factors, refer to Table 6.

In the univariate and multivariable logistic regression analysis (Table 7), we included six significant variables identified through comparison analysis, including age, gender, median of RR mean, NIHSS score at admission, eGFR, and hemoglobin. The gender, median of the RR mean, NIHSS score at admission, and eGFR showed consistent effects based on the odds ratios in both logistic regression analyses (gender: univariate OR = 0.306, adjusted OR = 0.166; median of RR mean: univariate OR = 0.997, adjusted OR = 0.989; eGFR: univariate OR = 0.985, adjusted OR = 0.981; Hb: univariate OR = 0.738, adjusted OR = 0.411). In the multivariable regression analysis, we found that the median of the RR mean (adjusted OR = 0.989, 95% CI = 0.982-0.997, *p*-value = 0.007), NIHSS score at admission (adjusted OR = 1.396, 95% CI = 1.135–1.717, *p*-value = 0.002), and hemoglobin concentration (adjusted OR = 0.411, 95% CI = 0.208–0.810, *p*-value = 0.010) were significantly associated with the 3-month behavioral functional outcome in ischemic stroke, and they were also considered as prognostic indicators. 

## 4. Discussion

The present study explored the relationship of HRV, neurologic function, and clinical factors with mortality and the 3-month behavioral functional in ischemic stroke. There were six major findings in our study. First, age was significantly lower in stroke survivors compared to the patients would did not survive. Second, female gender predominance was shown in patients with unfavorable outcome compared to favorable outcome. Third, NIHSS scores were significantly lower in stroke survivors compared to the patients who did not survive as well as in favorable outcome compared to unfavorable outcome. Fourth, hemoglobin levels were significantly correlated with behavioral functional outcome. Fifth, the median of the RR mean in patients with unfavorable outcome was significantly lower compared to in patients with favorable outcome. Sixth, the median of the RR mean was a potential prognostic indicator of 3-month behavioral functional outcome in ischemic stroke together with the NIHSS score at admission and hemoglobin levels. 

Stroke events and risk can be influenced by several modifiable and nonmodifiable risk factors, e.g., age, gender, and stroke-related disease history. In our study, the ischemic stroke patients were elderly and had a mean age of 70.9 years. This finding supports previous findings that stroke prevalence and mortality increase with the increasing of age. The increasing stroke risk factors and the impact on the cardiovascular system naturally happen in the elderly because of aging, which can increase the risk of ischemic stroke and mortality in ischemic stroke. A male predominance (56.9%) was indicated in our ischemic stroke cohort, which matches other studies from Jordan (56%), Egypt (54%), and Germany (58%) [13,14,15,16,17]. The higher risk of stroke in men could be explained by hormonal factors, higher smoking rates, and stress factors. Despite the male gender having a higher risk of stroke, the female gender was associated with unfavorable outcome in our study, which is concordant with several previous studies [18,19]. Some risk factors may contribute to this condition, such as atrial fibrillation and hypertension, which are common in women [8]. In our study, the one that may explain this situation is age. A previous study found that age caused the functional outcome to be worse [20].

In our study, the NIHSS score at admission was significantly associated with behavioral functional outcome. This finding is consistent with previous research, indicating that an early NIHSS is a strong prognostic factor for long-term functional outcome [21]. Additionally, NIHSS scores are widely recognized as universal predictors of functional outcome in ischemic stroke [22,23]. A previous study by Muir et al. showed the NIHSS score as being the best predictor for 3-month stroke outcome prediction, showing a positive association between NIHSS and mRS scores, highlighting the importance of stroke severity in functional outcome prediction [24]. NIHSS scores reflect the neurological function affected by brain infarction and are associated with the presence and location of vessel occlusion [25]. Higher NIHSS scores indicate more severe neurological deficits, which may reflect cerebral dysfunction. Therefore, early NIHSS scores may be used as a strong prognostic indicator for long-term functional outcome. However, NIHSS scores at admission were not significantly associated with mortality because of fewer deaths occurring in our study.

The other clinical factor that is significantly associated and becomes a potential prognostic indicator of long-term outcome is Hb in the present study. Similarly, Park et al. showed that a lower Hb range is related to poor outcome in acute ischemic stroke [26]. Moreover, low Hb has been linked to infarct volume expansion and infarct growth velocity acceleration [27,28]. However, several previous studies found low or high Hb to be associated with poor stroke outcome, mortality, and stroke recurrence [29,30,31]. Still, the association between Hb with stroke outcome and mortality faces uncertainty.

We also found that the eGFR was significantly different between the favorable and unfavorable groups, where the lower eGFR might indicate an unfavorable outcome. A previous study showed that low eGFR is associated with in-hospital death and at-discharge death or disability in ischemic stroke patients [32]. Patients with low eGFR indicated a reduced renal function, which increases the risk of development of cardiovascular diseases, including stroke. Renal function, represented by eGFR, also appeared as a significant independent prognostic indicator for long-term mortality over 10 years [33]. The eGFR cutoffs were found to have an association with poor outcome depending on the equation used to estimate GFR [34]. In contrast, Yang et al. showed no significant association between eGFR, mortality, and stroke outcome [35]. Those divulging results are complicated due to small sample sizes and not being population-based studies.

A common complication of stroke is autonomic dysfunction, which impairs the functional outcome and increases mortality [36]. Autonomic dysfunction in stroke is caused by the damage to the central autonomic network (CAN). The CAN consists of brain areas that play a critical role in emotional function, cognitive, and behavioral outcome, which are described as functional outcome in regard to stroke. The functional outcome of stroke was reported to have a correlation with ANS dysfunction and was measured by heart rate variability (HRV) [7]. In the present study, one of the HRV parameters showed statistically significant differences between the patients with poor behavioral functional outcome and good behavioral functional outcome, while patients with poor outcome had the lower median of RR mean. The median of RR mean at admission also was significantly associated with long-term functional outcome. These findings can be explained by ANS disruption in ischemic stroke. However, the RR mean association remains uncertain, because previous studies showed no significant association between the RR mean and stroke outcome [9,37]. Also, the TP, LF, HF, and LF/HF ratio in this study were lower in the unfavorable group than in the favorable group, but the difference was not statistically significant. In parallel, Tsai et al. also found the frequency-domain HRV parameter to showing less sensitivity to change and no significant correlation [10]. Yet, divergent results were found in several previous studies that explored the association between HRV parameters and behavioral functional outcome [7]. 

This study has several limitations. First, our study used a small cohort of Taiwanese ischemic stroke patients in a single hospital, which may cause selection bias and limit the generalization of our results to other stroke populations. The presence of diabetic patients, patients with atrial fibrillation, or medications also may have influenced the activity of the ANS. Second, we did not include a control group, preventing us from comparing the HRV as a prognostic factor in regard to ischemic stroke and healthy participants. Third, we focused only on specific HRV parameters, including the RR mean, TP, HF, LF, LF/HF, and LF%, thus limiting our ability to explore potential prognostic indicators from other HRV parameters, such as non-linear parameters. Fourth, only 58 patients were included in this study from among the 337 enrolled patients, which is a very small number, although one HRV parameter that was statistically significant was associated with long-term behavioral functional outcome. A study using large-scale data is still needed to explore and verify the association between HRV parameters and long-term functional outcome in ischemic stroke. Fifth, the functional outcome were measured only based on mRS scores. While the mRS score is a valid scale for representing the functional outcome in ischemic stroke, we acknowledge that using only the mRS score might not fully encompass all dimensions of functional status. Therefore, the suggested future research should enhance the comprehensiveness of evaluations by integrating additional assessment tools alongside the mRS.

## 5. Conclusions

Our study identified significant differences in age, NIHSS score at admission, hemoglobin, and stroke history between the survival and death groups. However, these variables did not show a significant correlation with mortality in this analysis. In contrast, variables such as age, gender, NIHSS score at admission, eGFR, hemoglobin, and the median of the RR mean varied significantly between the favorable and unfavorable outcome groups. Of these, the NIHSS score at admission, the median of the RR mean, and hemoglobin demonstrated a significant association with the 3-month functional outcome in ischemic stroke, suggesting their potential as useful prognostic indicators for long-term functional outcome. Meanwhile, frequency-domain HRV parameters did not show a significant relationship and were less sensitive in patients with poor outcome. Further clinical studies with larger datasets are necessary to explore the implications of HRV, neurological function, and clinical factors on mortality and behavioral functional outcome in ischemic stroke.

## Figures and Tables

**Table 1:** Baseline characteristic of all included patients.

Variables	Value
Age, mean ± SD	70.9 ± 13.31
Gender: Female/Male, number (%)	25 (43.1)/33 (56.9)
BMI: Obese, number (%)	31 (53.4)
Stroke Severity at admission	
Mild (NIHSS score <5)	11 (18.9)
Moderate (NIHSS score >5, ≤15)	27 (46.6)
Severe (NIHSS score >15)	20 (34.5)
Stroke Severity at discharge	
Mild (NIHSS score <5)	25 (43.1)
Moderate (NIHSS score >5, ≤15)	15 (25.9)
Severe (NIHSS score >15)	18 (31.0)
Infarct Side	
Left, number (%)	29 (50.0)
Right, number (%)	24 (41.4)
Bilateral, number (%)	5 (8.6)
Received tPa treatment, number (%)	28 (48.3)
HRV	
RR mean, median (IQR)	723.45 (265.83)
TP, median (IQR)	6.62 (2.66)
HF, median (IQR)	5.16 (2.77)
LF, median (IQR)	5.78 (2.55)
LF/HF ratio, median (IQR)	0.76 (0.93)
LF%, median (IQR)	71.42 (20.01)
Length of stay in hospital, mean ± SD	21.67 ± 18.15
Mortality status	
Survive/Death, number (%)	45 (77.6)/13 (22.4)
3-Month Functional Outcome	
Favorable (mRS score 0–2)/Unfavorable (mRS score 3–6)	28 (48.3)/30 (51.7)

The HRV parameters showed as median value. IQR is interquartile range. SD is standard deviation.

**Table 2:** Comparison of heart rate variability parameters and neurological function by mortality status in ischemic stroke.

Variables	Mortality		*p*-Value
	Survive n = 50	Death n = 8	
HRV			
RR mean, median (IQR)	715.93 (269.23)	784.03 (257.31)	0.755
TP, median (IQR)	6.62 (2.46)	6.71 (3.81)	0.638
HF, median (IQR)	5.08 (2.76)	5.76 (3.78)	0.343
LF, median (IQR)	5.66 (2.42)	5.95 (4.30)	0.603
LF/HF ratio, median (IQR)	0.78 (0.89)	0.62 (1.21)	0.387
LF%, median (IQR)	71.42 (18.27)	67.68 (29.57)	0.450
NIHSS score at admission, mean ± SD	11.54 ± 8.58	21.38 ± 6.97	**0.003**

The HRV parameters showed as median value. The *t*-test and Mann–Whitney u test were used when appropriate. The bold *p*-value indicates statistical significance at the 0.05 level (*p*-value < 0.05). IQR is interquartile range. SD is standard deviation.

**Table 3:** Comparison of clinical factors by mortality status in ischemic stroke.

Variables	Mortality		*p*-Value
	Survive n = 50	Death n = 8	
Age, mean ± SD	69.38 ± 12.73	80.38 ± 13.73	**0.029**
Female/Male, number (%)	21 (42.0%)/29 (58.0%)	4 (50.0%)/4 (50.0%)	0.671
BMI			0.330
Obese, number (%)	28 (56.0%)	3 (37.5%)	
non-obese, number (%)	22 (44.0%)	5 (62.5%)	
Infarct Side			0.570
Left, number (%)	24 (48.0%)	5 (62.5%)	
Right, number (%)	21 (42.0%)	3 (37.5%)	
Bilateral, number (%)	5 (10.0%)	0 (0.0%)	
Diseases History			
Hypertension/Other, number (%)	35 (70.0%)	7 (87.5%)	0.304
Drinking, number (%)	8 (16.0%)	0 (0.0%)	0.223
Smoking, number (%)	10 (20.0%)	1 (12.5%)	0.615
Diabetes mellitus, number (%)	22 (44.0%)	3 (37.5%)	0.730
Stroke, number (%)	5 (10.0%)	4 (50.0%)	**0.004**
Laboratory result at admission			
Hemoglobin (g/dL), mean ± SD	13.31 ± 2.23	11.53 ± 2.58	**0.044**
HbA1C (%), mean ± SD	6.46 ± 1.88	6.01 ± 0.57	0.508
Triglyceride (mg/dL), mean ± SD	126.35 ± 111.95	78.63 ± 32.49	0.240
Cholesterol (mg/dL), mean ± SD	181.71 ± 51.28	159.00 ± 68.69	0.274
K (mmol/L), mean ± SD	3.87 ± 0.42	4.11 ± 0.81	0.423
Bun (mg/dL), mean ± SD	17.66 ± 7.02	28.71 ± 17.78	0.153
Creatinin (mg/dL), mean ± SD	1.04 ± 0.52	1.49 ± 1.04	0.264
eGFR (mL/min/1.73 m^2^*)*, mean ± SD	78.33 ± 31.72	57.30 ± 26.60	0.081
Na (mmol/L), mean ± SD	138.70 ± 3.45	138.13 ± 4.29	0.674

The bold *p*-values indicate statistical significance at the 0.05 level (*p* < 0.05). The *t*-test, Mann–Whitney u test, and chi-square test were used when appropriate. SD is standard deviation.

**Table 4:** Univariate and Multivariable logistic regression analysis for mortality.

Variables	Univariate OR (95% CI)	*p*-Value	Adjusted OR (95% CI)	*p*-Value
NIHSS score at admission	1.134 (1.031–1.248)	**0.010**	1.109 (0.988–1.244)	0.079
Age	1.068 (1.003–1.138)	**0.039**	1.024 (0.944–1.111)	0.570
Stroke history	0.743 (0.548–1.008)	0.056	0.267 (0.041–1.736)	0.167
Hb	0.111 (0.021–0.588)	**0.010**	0.835 (0.565–1.235)	0.366

The bold *p*-values indicate statistical significance at the 0.05 level (*p* < 0.05).

**Table 5:** Comparison of HRV parameters and neurological function by the 3-month behavioral functional outcome in ischemic stroke.

Variables	3-Month Behavioral Functional Outcome		*p*-Value
	Favorable n = 28	Unfavorable n = 30	
HRV			
RR mean, median (IQR)	795.13 (278.40)	658.63 (272.50)	**0.049**
TP, median (IQR)	6.95 (2.11)	6.04 (3.48)	0.275
HF, median (IQR)	5.23 (1.92)	4.89 (4.11)	0.546
LF, median (IQR)	6.06 (1.98)	5.28 (3.72)	0.397
LF/HF ratio, median (IQR)	0.79 (0.89)	0.73 (1.07)	0.745
LF%, median (IQR)	71.02 (18.65)	71.60 (22.68)	0.587
NIHSS score at admission, mean ± SD	7.21 ± 4.83	18.20 ± 8.78	**<0.0001**

The HRV parameters showed as median value. The bold *p*-values indicate statistical significance at the 0.05 level (*p* < 0.05). The *t*-test and Mann–Whitney u test were used when appropriate. IQR is interquartile range. SD is standard deviation.

**Table 6:** Comparison of clinical factors by 3-month behavioral functional outcome in ischemic stroke.

Variables	3-Month Behavioral Functional Outcome		*p*-Value
	Favorable n = 28	Unfavorable n = 30	
Age, mean ± SD	66.25 ± 11.12	75.23 ± 13.88	**0.009**
Female/Male, number (%)	8 (28.6%)/20 (71.4%)	17 (56.7%)/13 (43.3%)	**0.031**
BMI			
Obese, number (%)	17 (60.7%)	14 (46.7%)	0.284
non-obese, number (%)	11 (39.3%)	16 (53.3%)	
Infarct Side			
Left, number (%)	10 (35.7%)	19 (63.3%)	0.109
Right, number (%)	15 (53.6%)	9 (30.0%)	
Bilateral, number (%)	3 (10.7%)	2 (6.7%)	
Diseases History			
Hypertension, number (%)	21 (75.0%)	21 (70.0%)	0.670
Drinking, number (%)	6 (21.4%)	2 (6.7%)	0.103
Smoking, number (%)	6 (21.4%)	5 (16.7%)	0.644
Diabetes mellitus, number (%)	15 (53.6%)	10 (33.3%)	0.120
Stroke, number (%)	1 (3.6%)	8 (26.7%)	**0.015**
Laboratory result at admission			
Hb (g/dL), mean ± SD	13.80 ± 1.52	12.38 ± 2.76	**0.018**
HbA1C (%), mean ± SD	6.50 ± 2.28	6.31 ± 1.12	0.698
Triglyceride (mg/dL), mean ± SD	139.68 ± 129.16	99.39 ± 71.73	0.155
Cholesterol (mg/dL), mean ± SD	184.61 ± 44.60	172.32 ± 62.16	0.399
K (mmol/L), mean ± SD	3.82 ± 0.36	3.97 ± 0.59	0.236
Bun (mg/dL), mean ± SD	17.40 ± 5.07	20.61 ± 12.16	0.273
Creatinin (mg/dL), mean ± SD	0.98 ± 0.30	1.21 ± 0.81	0.151
eGFR (mL/min/1.73 m^2^), mean ± SD	82.67 ± 26.61	68.68 ± 34.91	**0.022**
Na	138.32 ± 2.45	138.90 ± 4.35	0.532

The HRV parameters showed as median value. The bold *p*-values indicate statistical significance at the 0.05 level (*p* < 0.05). The *t*-test, Mann–Whitney u test, and chi-square were used when appropriate. SD is standard deviation.

**Table 7:** Univariate and multivariable logistic regression analysis for functional outcome.

Variables	Univariate OR (95% CI)	*p*-Value	Adjusted OR (95% CI)	*p*-Value
Age	1.060 (1.012–1.110)	**0.014**	0.980 (0.909–1.057)	0.597
Gender	0.306 (0.103–0.912)	**0.034**	0.166 (0.022–1.230)	0.079
RR mean	0.997 (0.994–1.000)	0.055	0.989 (0.982–0.997)	**0.007**
NIHSS score at admission	1.231 (1.109–1.367)	**<0.0001**	1.396 (1.135–1.717)	**0.002**
eGFR	0.985 (0.967–1.003)	0.103	0.981 (0.950–1.012)	0.219
Hb	0.738 (0.563–0.968)	**0.028**	0.411 (0.208–0.810)	**0.010**

Functional outcome referred to behavioral functional outcome. The bold *p*-values indicate statistical significance at the 0.05 level (*p* < 0.05). RR mean was represented as median value.

## Data Availability

Not available due to privacy and ethical reason.
